# Differential Responses of Neural Retina Progenitor Populations to Chronic Hyperglycemia

**DOI:** 10.3390/cells10113265

**Published:** 2021-11-22

**Authors:** Nicole Schmitner, Christina Recheis, Jakob Thönig, Robin A. Kimmel

**Affiliations:** Institute of Molecular Biology, Center for Molecular Biosciences Innsbruck (CMBI), Leopold Franzens University Innsbruck, 6020 Innsbruck, Austria; nicole.schmitner@uibk.ac.at (N.S.); christina.recheis@cranfield.ac.uk (C.R.); jakob.thoenig@student.uibk.ac.at (J.T.)

**Keywords:** retinal degeneration, regeneration, progenitor cell, diabetes, hyperglycemia, photoreceptors, neurod, Notch, zebrafish

## Abstract

Diabetic retinopathy is a frequent complication of longstanding diabetes, which comprises a complex interplay of microvascular abnormalities and neurodegeneration. Zebrafish harboring a homozygous mutation in the pancreatic transcription factor *pdx1* display a diabetic phenotype with survival into adulthood, and are therefore uniquely suitable among zebrafish models for studying pathologies associated with persistent diabetic conditions. We have previously shown that, starting at three months of age, *pdx1* mutants exhibit not only vascular but also neuro-retinal pathologies manifesting as photoreceptor dysfunction and loss, similar to human diabetic retinopathy. Here, we further characterize injury and regenerative responses and examine the effects on progenitor cell populations. Consistent with a negative impact of hyperglycemia on neurogenesis, stem cells of the ciliary marginal zone show an exacerbation of aging-related proliferative decline. In contrast to the robust Müller glial cell proliferation seen following acute retinal injury, the *pdx1* mutant shows replenishment of both rod and cone photoreceptors from slow-cycling, neurod-expressing progenitors which first accumulate in the inner nuclear layer. Overall, we demonstrate a diabetic retinopathy model which shows pathological features of the human disease evolving alongside an ongoing restorative process that replaces lost photoreceptors, at the same time suggesting an unappreciated phenotypic continuum between multipotent and photoreceptor-committed progenitors.

## 1. Introduction

Diabetic retinopathy (DR) is the leading cause of vision loss in working-age adults [1], and the global burden of visual impairment and blindness attributable to DR was projected to rise to 3.2 million affected people in 2020 [2]. Neural tissues such as the brain and retina depend entirely on glucose as the major fuel driving energy metabolism [3]. In normal retina, the blood–retinal barrier maintains a stable milieu even during changing plasma glucose concentrations. When plasma glucose is pathologically high over prolonged periods of time, as in diabetes, these regulatory mechanisms are disrupted. As a result, the retina becomes extremely vulnerable to tissue damage, as is manifested in diabetic retinopathy [4]. While vascular features (i.e., capillary degeneration, increased vascular permeability, and neovascularization) are prominent in DR, the pathological importance of accompanying neural damage is increasingly appreciated [5,6]. Retinal neurodegeneration may even precede and exacerbate diabetes-induced vascular changes, making neuroprotection an emerging potential therapeutic target [6]. Although all cell types in the retina are affected by diabetes, injury to the outer retina, comprising photoreceptors and retinal pigment epithelium (RPE), is proposed to have a special role for development of retinal vascular and neural lesions [7].

The anatomy and function of the retina are well conserved between zebrafish and mammals. While nocturnal rodents have a rod dominated retina [8], zebrafish have a relatively cone-rich retina similar to the human macula, and therefore represent an important complementary model to extend our knowledge about retinal pathogenesis during DR. In both mammalian and zebrafish retina, Müller glia provide essential mechanical and metabolic support to all retinal neurons through cellular projections that extend laterally as well as along the apical–basal axis [9]. They can sense and react to cell stress or damage with a response that is protective or harmful, depending on the extent of injury. Under pathological conditions, Müller glia cells in mammals rarely proliferate, and when they do, they establish a glial scar [10]. Unlike the mammalian retina, the zebrafish retina has an innate capacity for regeneration. Upon acute retinal injury, zebrafish Müller glia become activated but switch from a gliotic stage and change their transcriptional program to acquire stem cell characteristics [11]. This is followed by asymmetric division which allows for self-renewal and generates multipotent progenitor cells that can actively divide and regenerate all major retinal cell types [11,12]. Understanding the mechanisms that resolve gliosis and support regeneration in zebrafish might prove useful to elaborate for protective or replacement therapies in humans.

Zebrafish is an attractive model system for studying metabolic diseases to improve the understanding of pathologies and progression and to discover and characterize new diagnostic and therapeutic targets. As a model organism, zebrafish offer the advantages of ease of maintenance, a relatively short reproduction time and amenability to genetic and pharmacological manipulation. Importantly, the cornerstones of mammalian glucose homeostasis are conserved in zebrafish. They possess all main metabolic organs including liver, pancreas and adipose tissue, initiate gluconeogenesis during fasting [13,14], store and catabolize glycogen [15] and respond to insulin injections [16]. Additionally, loss of β-cells in larval and adult fish leads to hyperglycemia, mimicking a diabetic state [17,18]. Mutations in genes affecting β-cell development in zebrafish result in phenotypes resembling those associated with the human diabetic condition. For example, *pdx1* is linked to genetic forms of diabetes and is associated with increased susceptibility to type-2 diabetes in humans [19]. The *pdx1* mutant diabetic zebrafish genetically resembles human neonatal diabetes in that homozygous mutants show early onset, persistent hyperglycemia, along with reduced insulin and reduced β-cells [20].

Adult zebrafish models of diabetic retinopathy have been scarce but have highlighted similarities between mammals and zebrafish [21]. Previous studies addressing the effects of hyperglycemic conditions on the neural retina have mostly relied upon immersion in glucose solutions, which can only be maintained for several weeks. Retinal changes included decreased thickness of the inner plexiform layer (IPL) and inner nuclear layer (INL) [22], pathological changes in cone photoreceptors and altered cone-mediated electroretinogram responses [23,24]. Ablation of beta cells through streptozotocin injection induced hyperglycemia, that was limited to three weeks due to beta cell regeneration, and led to thinning of the IPL and photoreceptor layer (PR) [25]. In the genetic *pdx1* mutant diabetic zebrafish, hyperglycemia is maintained from larval stages through adulthood [20,26], enabling long-term studies. Reports of the retinal phenotype of *pdx1* mutants focused on the vasculature and neurodegenerative changes, while loss of photoreceptors was also observed [26,27]. Zebrafish are known to possess the capacity to regenerate retinal neurons following injury [28], but a reparative response to damage from chronic metabolic dysregulation has not been described.

Using the *pdx1* diabetic zebrafish model, we now characterized the neuronal injuries caused by prolonged hyperglycemia and defined retinal cell-type specific responses. A low-grade injury was suggested by the absence of a detectable increase in cell death and lack of immune cell infiltration. We examined mitosis in progenitor populations and uncovered changes in proliferative capacity that are age- and hyperglycemia-dependent. Furthermore, newly generated cones arise in the absence of Müller glia activation, suggesting an unappreciated plasticity of the rod-lineage progenitor cell. Overall, these studies indicate that in the zebrafish retina under persistent hyperglycemic conditions, chronic cell loss is accompanied by an ongoing restorative response to replenish lost photoreceptors from an endogenous progenitor population.

## 2. Materials and Methods

### 2.1. Animal Husbandry

The zebrafish used were previously described *pdx1* mutants [20], and maintained according to standard procedures as homozygous, heterozygous and wild type siblings. The studies used non-transgenics and fish containing the following previously published transgenes: neurod:GFP [29], pax6b:dsRed [30], Tp1:GFP [31] and Tp1:hmgb1-mCherry [32]. In total, 36 control animals and 29 *pdx1* mutants were used (8 control and 5 *pdx1* mutants at 3 months post-fertilization (mpf), 16 controls and 13 *pdx1* mutants at 6 mpf and 12 controls and 11 *pdx1* mutants at the age of 8 to 10 mpf. Animals were staged by month as indicated. Genotyping was performed as previously described [20]. All procedures were approved by the Austrian Bundesministerium für Wissenchaft und Forschung (GZ BMWFW-66.008/0023-WF/V/3b/2016, GZ BMWFW-66.008/0018-WF/V/3b/2017, and GZ. 2020-0.282.289). 

### 2.2. Histology

Animals were fasted overnight to eliminate confounding effects of variable feeding behavior on glucose measurements. They were then dark adapted for two hours, which contracts pigments to the basal RPE, in order to minimize pigment granule interference with visualization of photoreceptors [33]. The animals were then euthanised on ice and glucose was measured. Subsequently, the eyes were dissected, fixed in 4% paraformaldehyde (PFA) overnight, equilibrated in 30% sucrose overnight and embedded in OCT medium. Cryosections (18 µm) were cut on a Reichert-Jung Frigocut 2800E and mounted on silane-coated slides. Feulgen staining [34] was performed on cryosections after dehydration in ethanol overnight. The slides were examined with a Leica DM5000B microscope with a 40x objective and imaged with a Leica DF490 digital camera using the Leica application suite (Version 4.8).

### 2.3. Immunohistochemistry

Before immunolabeling, cryosections were dried at room temperature then incubated in cold methanol for 10 min, followed by 10 min incubation in 1%DMSO/1%TritonX-100/PBS. The slides were washed in PBS-0.1% Tween and incubated in blocking solution (PBS + 1% BSA, 5% sheep serum, 0.1% TritonX-100) for at least 1 h. The following primary antibodies were used: GFAP (Zrf1, 1:1000, ZDB-ATB-081002-46, ZIRC, Eugene, OR, USA), L-plastin (1:100, GTX124420, GeneTex, Irvine, CA, USA) and dsRed (1:200, 632496, Takara Bio Clontech, Saint-Germain-en-Laye, France). Alexa-conjugated secondary antibodies were used at dilutions of 1:500 or 1:1000 in blocking buffer. Cones were stained with CF568 Lectin PNA (1:100, Cat.No. 29061, BioTrend, Cologne, Germany). Slides were counterstained with DAPI to label nuclei. Sections were imaged on a Zeiss LSM700 with a 40× objective or a Zeiss Axio Observer.Z1 with a Yokogawa CSU-X1 spinning disk using a 25× objective, and processed in ImageJ. Regions were selected to include the CMZ (“peripheral”) or to include the optic nerve and/or directly adjacent tissues (“central”). The dsRed fluorophore was detected by antibody staining; for all other transgenes, native fluorescence is shown and was considered for analysis.

### 2.4. EdU Labeling

EdU staining was performed to label proliferating cells. To this end, *pdx1* mutants and age-matched controls were injected intraperitoneally with 20 µg/g bodyweight EdU (5-ethynyl-20-deoxyuridine). Fish were sacrificed after two days, glucose was measured and eyes were fixed in 4% PFA and cryosectioned. EdU detection was performed according to manufacturer’s instruction using Click iT EdU Alexa 488 (C10337, Invitrogen, Waltham, MA, USA) or Sulfo-Cy5 (A3330, Lumiprobe, Hannover, Germany). Sections were then further processed for antibody staining and counterstained with DAPI.

### 2.5. Terminal Deoxynucleotidyl Transferase-dUTP Nick End Labeling (TUNEL) Assay

Cryosections from *pdx1* mutant and age matched control retinae were used to evaluate cell death. TUNEL was performed using In Situ Cell Death Detection Kit, Fluorescein (11684795910, Roche, Basel, Switzerland) according to the manufacturer’s protocol. DAPI was used for counterstaining.

### 2.6. In Situ Hybridisation

In situ hybridization was performed with digoxigenin labeled antisense RNA probes (DIG RNA Labelling Mix, 11277073910, Roche, Basel, Switzerland) and α-Digoxigenin-AP antibody (1:1500, 11093274910, Roche, Basel, Switzerland) as described in [35], with some modifications. Briefly, the *pax6a* antisense RNA probe [36] was generated after linearizing the corresponding plasmid using T7 RNA Polymerase. Sections were incubated in cold methanol for 10 min, followed by 10 min incubation in 1% DMSO/1% TritonX-100/PBS and post-fixation in 4% PFA. Sections were then acetylated for 10 min and hybridized with DIG-labeled probe overnight at 68 °C. Post-hybridization washes were followed by an RNase treatment (20 μg/mL RNase A in 400 mM NaCl, 10 mM Tris (pH 7.5), 5 mM EDTA). After blocking, sections were incubated overnight in blocking solution containing anti-DIG antibody. Color was developed using NBT/BCIP. Sections were imaged on a Leica DM6000 using a 40× objective.

### 2.7. Blood Glucose Measurement

Adult zebrafish were fasted overnight and euthanized on ice for measurements under fasting conditions. Blood glucose was measured according to previously published methods [37]. In brief, blood from a pectoral girdle incision was directly applied to a Freestyle Lite glucose test strip, and glucose readings were obtained from the Freestyle Lite glucose meter (Abbott, Chicago, IL, USA) as per manufacturer’s instructions. The detection range of the instrument is between 20 mg/dL (LO) and 500 mg/dL (HI). Therefore, LO readings were recorded as 20 and HI readings as 500 mg/dL. We could assure sustained high glucose levels in analyzed *pdx1*^−/−^ mutant zebrafish from 3 to 10 months of age (Figure S1).

### 2.8. Cell Counting

Counting of TUNEL positive cells was performed manually on at least three sections per individual with a field of view comprising 320.09 µm × 320.09 µm. The length of the retina in the field of view was then measured and the amount of cells per 100 µm was calculated.

For quantification of L-plastin cells, ‘inner retina’ refers to GC, IPL and INL, ‘outer retina’ includes OPL, ONL, PL and RPE. Two sections per individual fish with a field of view comprising 266.24 µm × 266.24 µm were analyzed. The length of retina in the field of view was then measured and the amount of cells per 100 µm was calculated.

EdU positive cells were manually counted on whole sections and assigned to layers with at least three sections per individual. For the estimated cell number in the ONL, DAPI stained nuclei were counted in three regions/section of 50 µm length. The length of the whole neural retina was measured from a low-magnification image and DAPI positive cell number was then extrapolated from the 50 µm long region to the whole length of the retina. These values were used to calculate the proportion of EdU positive cells in the ONL.

Neurod:GFP positive cells in the INL were counted with the ImageJ plugin StarDist using the built-in versatile model for fluorescent nuclei [38] in 50 µm long regions of at least three sections per individual. For quantitating samples with neurod:GFP positive rods and cones, a field of view comprised a region of 266.24 µm × 266.24 µm, and at least three sections per individual were analyzed.

### 2.9. Statistics and Data Analysis

Counts per individual were averaged, data were analyzed and graphs were generated using Graphpad Prism (Version 9.2.0). The statistical significance of differences between controls and mutants was determined using either two-tailed, unpaired *t*-test to compare two groups or two-way ANOVA when considering genotype and age.

## 3. Results

### 3.1. In pdx1 Mutants, Photoreceptor Degeneration Is Not Accompanied by Signs of Acute Injury

**Figure 1:**
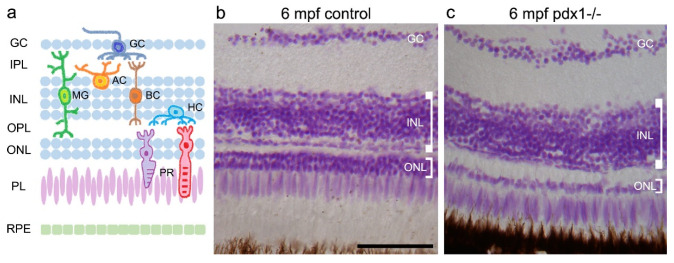
Pathology in the neural retina of *pdx1*^−/−^ mutants at 6 mpf. (**a**) Schematic showing the locations of the cell types of the neural retina. Feulgen staining of cryosections of control (**b**) and *pdx1* mutant (**c**) zebrafish. Scale bar: 50 µm. (GC, ganglion cell layer; IPL, inner plexiform layer; INL, inner nuclear layer; OPL, outer plexiform layer; ONL, outer nuclear layer; PL, photoreceptor layer; RPE, retinal pigment epithelium; AC, amacrine cell; MG, Müller glial cell; BC, bipolar cell; HC, horizontal cell; PR, photoreceptor).

Similar to other vertebrates, including mammals, the zebrafish retina contains three nuclear and two synaptic layers. The cell bodies of neural bipolar, horizontal and amacrine cells, as well as the Müller glial cells, are situated in the inner nuclear layer (INL), while photoreceptors reside in the outer nuclear layer (ONL) and adjacent photoreceptor layer (PL), with their light-collecting outer segments supported by processes from the underlying retinal pigment epithelium (RPE). Axonal and dendritic processes extend from these cells and make connections within the intervening inner and outer plexiform layers (IPL and OPL, Figure 1a). Histological examination of retina sections from age-matched *pdx1* mutants and controls revealed that the changes previously reported at older stages [26] are detectable by 6 mpf (Figure 1b,c). The ONL, containing rod and UV cone nuclei, was reduced to 1–2 cell layers, and photoreceptor outer segments were truncated (*n* = 3/3) (Figure 1c and Figure S2a).

**Figure 2:**
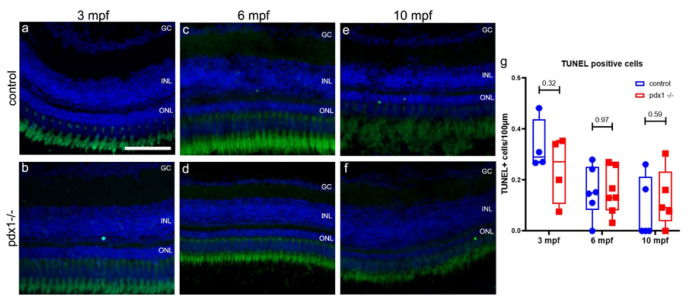
Hyperglycemia does not lead to increased cell death in the neural retina of *pdx1*^−/−^ mutants. Cryosections of retina at 3 (**a**,**b**), 6 (**c**,**d**) and 10 (**e**,**f**) mpf from control and *pdx1*^−/−^ mutants labeled by TUNEL staining (green) and counterstained with DAPI (blue). (**g**) Quantification of TUNEL positive nuclei in all layers of the retina. Box plot extends from 75% to 25%, showing all data points, line indicates median, *n* = 4–7 fish per time point. Scale bar: 50 µm. (GC, ganglion cell layer; INL, inner nuclear layer; ONL, outer nuclear layer).

**Figure 3:**
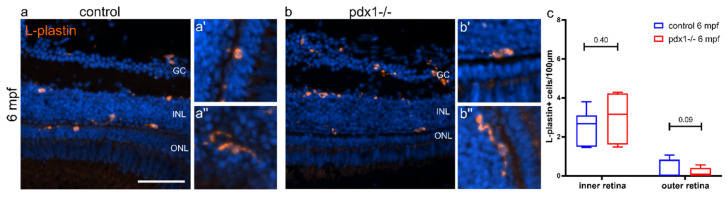
Immunolocalization of immune cells in the retina of control and *pdx1*^−/−^ mutant zebrafish. Cryosections of 6 mpf control (**a**) and *pdx1*^−/−^ mutant (**b**) retinas stained for L-plastin (orange) and counterstained with DAPI (blue). Close-up images of compact and round (**a′**,**b′**) versus elongated (**a**″,**b**″) L-plastin labeled cells found in control (**a′**,**a**″) and *pdx1*^−/−^ mutants (**b′**,**b**″). (**c**) Quantification of L-plastin positive cells in the inner and the outer retina (for details see Materials and Methods). Box plot extends from 75% to 25%, whiskers showing minimum and maximum, line indicates median, *n* = 7 controls, 6 *pdx*^−/−^. Scale bar: 50 µm. (GC, ganglion cell layer; INL, inner nuclear layer; ONL, outer nuclear layer;).

The extensive cell death that follows acute retinal injuries manifests in enhanced apoptosis [28]. To assess if a detectable increase in cell death correlates with the reduced numbers of rods and cones in the *pdx1* mutant, we performed TUNEL staining on cryosections from *pdx1* mutants and age-matched controls. We used adult stages representing the early disease state (3 mpf), established disease (6 mpf) and a prolonged disease state (10 mpf). Analysis at all time points revealed only few apoptotic cells, with no significant difference between wild types and mutants (Figure 2). Antibody staining for active caspase-3 confirmed these findings (data not shown). It has been reported that degeneration of photoreceptors is accompanied by an inflammatory response from microglia [39,40]. While elevated levels of cell death were not detected, we investigated whether there was a low-grade injury signal sufficient to attract immune cells. In 6 mpf control and *pdx1* mutant samples, microglia, as labeled by an antibody to L-plastin (lymphocyte actin-binding protein), were found in similar numbers within the inner retinal layers while there was a reduction in L-plastin stained cells in the outer retinal layers in *pdx1* mutants, but this was not significant (Figure 3a–c). Cell shape in both controls and mutants ranged from compact and ovoid to complex, extended morphologies (Figure 3a′,a″,b′,b″). Overall, we could not detect increased or activated immune cells in the central or peripheral regions of the retina of 3, 6 and 10 mpf *pdx1* mutant fish. As we did not observe increased cell death or an accompanying inflammatory response, prolonged hyperglycemia apparently does not cause acute damage, but is rather a continuous low-grade injury.

### 3.2. Changes in Retinal Proliferation Due to Aging and Hyperglycemia

In the constantly expanding zebrafish retina, stem cells in the ciliary marginal zone (CMZ) produce all neurons except rods, while rods are generated from Müller glia-derived rod progenitors [9,12]. To determine whether hyperglycemia-induced suppression of proliferation contributes to the reduced photoreceptor cell numbers, we examined proliferation by injecting EdU into fish aged 3, 6 and 10 mpf, and harvested the eyes two days later. The labeling time of 48 h was used to identify proliferation events occurring over that extended time period, to see dividing cells as well as their progeny.

**Figure 4:**
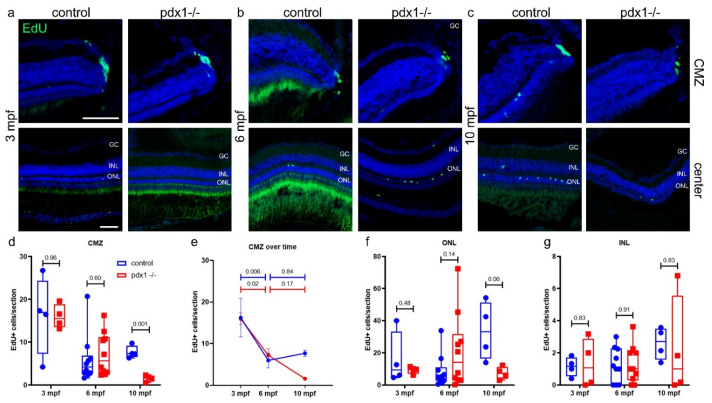
Chronic hyperglycemia impacts proliferation in progenitor populations. Cryosections of control and *pdx1*^−/−^ retinas at 3 mpf (**a**), 6 mpf (**b**) and 10 mpf (**c**). Cryosections were stained for EdU to identify cells that underwent proliferation (green) and counterstained with DAPI (blue). (**d**,**e**) Quantification of EdU positive cells in the ciliary marginal zone (CMZ) depicted in a box plot (**d**) and the same data shown as a line plot over time (**e**). Quantification of EdU positive cells in the ONL (**f**) and the INL (**g**) at 3, 6 and 10 mpf. Box plot extends from 75% to 25%, showing all data points, line indicates median. *n* = 4–10 fish per time point. Scale bar: 50 µm. (GC, ganglion cell layer; INL, inner nuclear layer; ONL, outer nuclear layer).

The peripheral CMZ contained a cluster of proliferative cells, and consistent with previous reports, CMZ proliferation declined with age [41] (Figure 4a–e). In comparing CMZ proliferation between controls and *pdx1* mutants, EdU+ cell counts were similar at the early adult stages of three and six months (Figure 4d). At 10 mpf, *pdx1* mutants showed a significantly decreased rate of proliferation compared to the controls (Figure 4d). When considering changes that occurred with aging, we observed that proliferative activity was higher at 3 mpf as compared to 6 and 10 mpf in the controls (Figure 4e). Proliferative activity dropped similarly between 3 and 6 mpf in the *pdx1* mutants, while the number of EdU positive CMZ cells in mutants further decreased when comparing 6 mpf and 10 mpf, although this was not a statistically significant reduction (Figure 4e).

In the ONL, where rod progenitors are located, proliferation is readily detectable (Figure 4a–c,f). The ONL in *pdx1* mutants appeared thinner at 6 and 10 mpf, and calculating the absolute amount of cells in the ONL yielded a significant decrease at these time points (Figure S2a). To determine whether the changes in EdU+ cells depended on the total number of ONL cells, we examined the proportion of EdU+ cells in relation to ONL cell number. This analysis revealed a significant increase in ONL proliferation in the mutants at 6 mpf (Figure S2b). Interestingly, in 6 mpf *pdx1* mutants, we could differentiate between a mild phenotype with few proliferating cells in the ONL and a mildly reduced cell number in the ONL (thickness of 3–5 cell layers) (Figure S3a), and a severe phenotype with increased proliferating cells in the ONL associated with severely decreased cells in the ONL (thickness of 1–2 cell layers) (Figure 4b,f and Figure S3b). The phenotype severity did not correlate with blood glucose levels as measured at the time of tissue harvest (Figure S3c). Examination of glial fibrillary acidic protein (GFAP) staining as an indicator of Müller glia did not show a change in intensity or distribution to reflect the injury or proliferative response (Figures S3a,b and S4). At 10 mpf, *pdx1* mutant samples showed a trend towards decreased proliferation in the ONL as compared to controls (*n* = 4), which did not reach statistical significance (Figure 4f and Figure S2b).

Cells of the INL are generally post-mitotic, with the exception of slowly dividing Müller glia [9]. Enhanced proliferation in the INL in response to injury suggests a regenerative response that is initiated by Müller glia cells [42]. In the INL, proliferation was low and stable over time in the controls, and this was not different in the mutants (Figure 4g). In only one mutant sample of all time points examined (*n* = 18), harvested at 10 mpf, was a marked increase in proliferative cells in the INL observed (Figure 4g and Figure S3e), which coincided with a severely reduced ONL thickness (1–2 cell layers) but not with the degree of hyperglycemia (Figure S3e,f). Similar to the 6 mpf samples, the activated proliferation was not accompanied by a detectable upregulation of GFAP expression (Figure S3d–f).

Overall, new cells are generated with equivalent frequencies at early adult stages, but stem cells in the diabetic *pdx1* mutants show reduced proliferation at later stages. This suggests that the decreased proliferative capacity in the retina seen with normal aging is exacerbated by hyperglycemia. Furthermore, in the majority of mutants examined (*n* = 17/18) there was no increase in proliferation in the INL that would be suggestive of activation of Müller glial cells.

### 3.3. Müller Glia Do Not Show Signs of Activation

Reports suggest that Notch signaling plays a critical role in the transition of Müller glia from a quiescent to regenerative state, although the literature contains examples of injury- and regeneration-associated up- as well as down-regulation of Notch signaling [11,12,43]. In mice and human cells in culture, Notch signaling contributes to the Müller glia gliotic (scarring) response to injury [44]. To address the potential role of Notch signaling in the response to metabolic injury, and given the differing responses reported depending on the model investigated, we first examined the temporal dynamics of Notch signaling during post-embryonic development using Tp1:GFP;Tp1:hmgb1-mCherry Notch reporter double transgenics [31]. In these fish, after endogenous Notch expression is downregulated, GFP remains visible for a short time due to protein perdurance, while the nuclear-targeted hmgb1-mCherry fluorescent tag will remain detectable long beyond the duration of active Notch signaling in slow-cycling cells [45].

**Figure 5:**
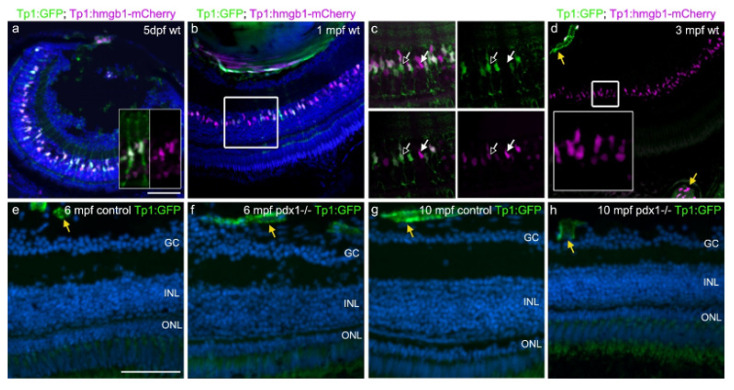
Notch signaling is not re-activated during chronic hyperglycemia. (**a**–**d**) Tg(tp1:GFP;tp1:hmgb1-mCherry) double transgenic 5 dpf larvae (**a**) and adults (**b**–**d**) showed gradual loss of GFP expression in the INL over time, while hmgb1-mCherry expression labeled cells in the INL through adulthood. In some GFP-positive cells at 5 dpf, projections extend along the apical–basal axis (**a**, inset). In (**c**), which shows a close-up of the boxed region in (**b**), GFP and mCherry variably overlap in INL nuclei. At 3 mpf, INL cells show variable levels of mCherry, and are GFP negative (**d**, inset). GFP and mCherry are coexpressed in retinal and choroidal vessels (**d**, yellow arrows). (**e**–**h**) In 6 mpf (**e**,**f**) and 10 mpf (**g**,**h**) control and *pdx1^−/−^* mutants, GFP expression via notch-responsive elements cannot be detected in the neural retina. Expression is limited to retinal and choroidal vessels (yellow arrows). Sections were counterstained with DAPI (blue). Scale bar: 50 µm. (GC, ganglion cell layer; INL, inner nuclear layer; ONL, outer nuclear layer).

In larvae at five days post fertilization (dpf), we found overlapping GFP and nuclear mCherry expression in retinal neurons in the INL (Figure 5a). Elongated nuclei in the center of the INL were labeled strongly with GFP. As revealed by the cytoplasmic GFP, their cell morphology was consistent with Müller glia, with GFP-labeled processes extending to the GCL, ONL and PR layers (Figure 5a, inset). Cells with a rounded nuclear morphology showed only weak or no GFP expression (Figure 5a). At 1 mpf, GFP and mCherry labeled cells were detected in the INL, with individual cells varying in their relative levels of GFP and mCherry (Figure 5b,c). In double-labeled cells, GFP-labeled processes extended to the ONL and PR layers (Figure 5c). Strong expression of both transgenes was suggestive of ongoing Notch signaling (Figure 5c, black arrow). Cells with nuclear mCherry and an absent or weak GFP signal suggested that the cell previously responded to Notch (Figure 5c, white arrow, insets). As demonstrated in Medaka, Notch signaling promotes neuronal differentiation in the INL and is active longer in Müller glia cells [46].

By 3 mpf, GFP expression was no longer detected within the INL while mCherry+ nuclei remained, reflecting that these cells had experienced Notch signaling during their developmental history (Figure 5d). Levels of nuclear mCherry varied from bright to dim, indicating the mix of cell lineages, including progenitors that have divided, as well as more quiescent cells. Expression of both cytoplasmic GFP and nuclear mCherry were readily detectable in retinal and choroid vessels at this stage (Figure 5d, yellow arrows), confirming that both transgenes are active.

These studies suggest that Notch signaling remains active in the vasculature but is not signaling in the healthy neural retina at adult stages. As upregulation of Notch pathway components has been detected in response to injury [12,47], we examined whether increased Notch signaling is a feature of the response to chronic hyperglycemia in *pdx1* mutants. At 6 and 10 mpf, in *pdx1^−/−^* mutants as in controls, Tp1-driven GFP expression was not observed in the zebrafish neural retina, while it was detectable in the retinal vasculature (Figure 5e–h yellow arrows).

**Figure 6:**
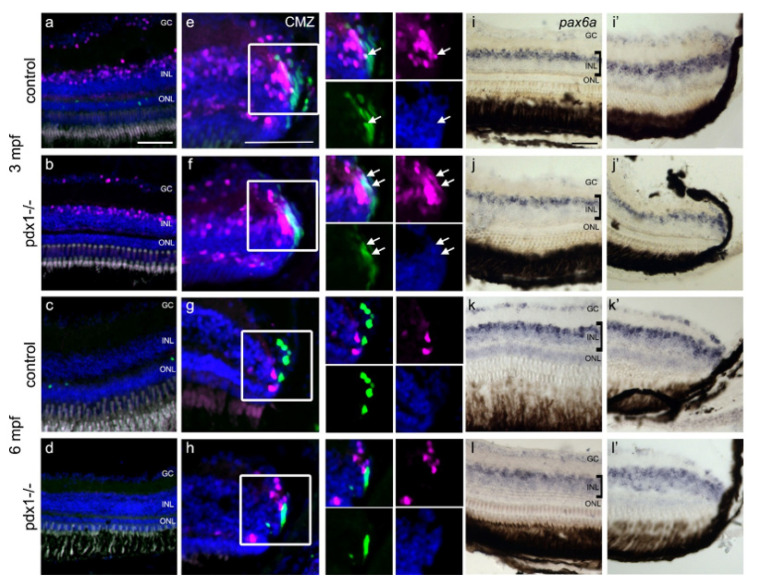
*pax6*-expressing progenitors are not increased in chronic hyperglycemia. Tg(pax6b:dsRed) labeled cells (magenta) in the INL (**a**,**b**) and the CMZ (**e**,**f**) in 3 mpf control and *pdx1^−/−^* mutant retinas. Proliferating cells are labeled through EdU incorporation (green). In 6 mpf controls (**c**,**g**) and *pdx1^−/−^* mutants (**d**,**h**), pax6b:dsRed expression was not observed in the central area of the retina and was limited to few cells in the CMZ. In situ hybridization for *pax6a* stained cells in the basal portion of INL in the central area of the retina (**i**–**l**) and in the CMZ (**i′**–**l′**) in controls (**i**,**i′**,**k**,**k′**) and *pdx1^−/−^* mutants (**j**,**j′**,**l**,**l′**) at 3 mpf and 6 mpf. Scale bar: 50 µm. (GC, ganglion cell layer; INL, inner nuclear layer; ONL, outer nuclear layer).

Through an asymmetric division, activated Müller glia cells give rise to progenitors that depend on pax6 for further rounds of proliferation [11]. To rule out that undetected proliferation has yielded such Müller glia-derived multipotent progenitor cells, we examined expression of *pax6*. Zebrafish possess two paralogous *pax6* genes, and during regeneration, *pax6b* is expressed in the earliest proliferating progenitors while *pax6a* is required for later neuronal progenitor cell divisions [48]. *Pax6b* was examined using the pax6b:dsRed transgene [30] in conjunction with EdU labeling for proliferation. In 3 mpf samples, dsRed expression was observed in the GCL and in non-proliferating cells in the INL in both controls and mutants (Figure 6a,b). In the CMZ at 3 mpf, dsRed was detected in cells next to the proliferating cell niche in wild type and *pdx1* mutant fish (Figure 6e,f). In both controls and mutants, a few cells showed overlapping expression of EdU and pax6b:dsRed (Table 1), a pattern indicating progenitors exiting the stem cell compartment [12]. At 6 mpf, pax6b:dsRed was no longer detected in the central INL (Figure 6c,d). Expression was reduced to a few cells adjacent to the proliferating cells of the CMZ, and overlap with EdU was only rarely detected (Figure 6g,h, Table 1), consistent with reduced rates of new cell addition after the rapid growth of early adulthood [41]. Thus, the pax6b:dsRed transgene was readily detectable in progenitor cells at the CMZ, while no EdU+/pax6+ cells were detected in the INL in mutants to indicate generation of proliferative progenitors.

To assess expression of *pax6a*, we performed in situ hybridization. *Pax6a* transcript was detected in the GC and in the basal portion of the INL, consistent with the location of amacrine cells. The expression pattern in the INL (Figure 6i–l, left panel), as well as in the CMZ (Figure 6i′–l′), was unchanged when comparing controls and hyperglycemic mutants.

### 3.4. Neurod-Expressing Cells Replace Lost Photoreceptors

The relatively stable but reduced PR number over time in *pdx1* mutants (Figure S2), in the face of continuing metabolic dysregulation, suggests that photoreceptors may be restored as they are lost, at least to some extent. Rod photoreceptors are supplemented throughout the zebrafish-lifespan through a population of rod progenitor cells that arise in the INL, proliferate and move into the ONL, where they further proliferate before differentiating [49]. While *pax6* is initially expressed by multipotent retinal progenitors derived from Müller glia cells and those arising from the CMZ, this expression is transient, and neurod expression characterizes cells fated to become photoreceptors [12,49,50]. In adult zebrafish, neurod is expressed in amacrine cells and bipolar cells of the INL and GCs, in addition to developing rod and cone photoreceptors [50,51,52].

To distinguish progenitor populations and eventual fates, we examined how fate specification is manifested by pax6b:dsRed and neurod:GFP transgene expression [51], which also allows for short-term lineage tracing. In the larval retina (5 dpf), neurod:GFP+ cells were found adjacent to the pax6b:dsRed+ cells of the CMZ, and showed weak dsRed expression (Figure S5a,b, black arrow). Neurod:GFP+ cells located further from the CMZ do not express dsRed (Figure S5b, white arrow). In the central retina, dsRed and GFP mark distinct neuronal types within the basal portion of the INL (Figure S5c). DsRed is not detected in the photoreceptor layer, while GFP labels ONL cells with a range of strong to weak expression (Figure S5a,c, right panel). At 1 mpf, pax6b:dsRed cells formed a cluster at the CMZ, while neurod:GFP cells were found in the adjacent region, interspersed with dsRed-positive cells (Figure S5d,e). In the central retina, we observed vertical clusters of cells extending from the INL into the ONL that showed an elongated morphology and were strongly GFP positive, but dsRed negative (Figure S5f). This pattern was similar to what has been previously described for rod progenitors that proliferate and migrate from the INL to the ONL, where they exit the cell cycle and complete differentiation [49]. Within the ONL, stronger GFP expression labeled clusters of photoreceptors, consistent with an origin from progenitors that amplify within the ONL (Figure S5d yellow arrows). Therefore, the neurod:GFP transgene expression indicates differentiating photoreceptors newly arising from progenitor cells which no longer express *pax6*.

**Figure 7:**
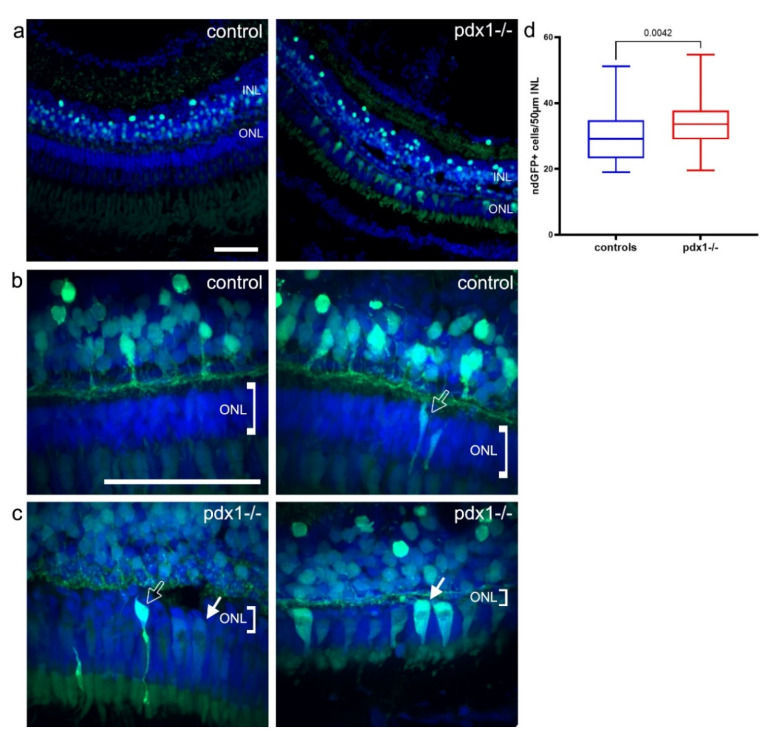
Photoreceptors are restored from *neurod*-expressing progenitors. (**a**–**c**) In neurod:GFP transgenics, strong GFP is detected mainly in the INL in controls, and in the INL and ONL of *pdx1* mutants (**a**). In controls, neurod:GFP labeled rod-shaped cells in the ONL were observed. In *pdx1*^−/−^ mutants, neurod:GFP labeled rod and cone shaped cells in the ONL in most mutants (**b**,**c**, see Table 2). Sections were counterstained with DAPI (blue). A significant increase in GFP positive cells in the INL was detected in *pdx1*^−/−^ mutants (**d**). Box plot extends from 75% to 25%, whiskers showing minimum and maximum, line indicates median, *n* = 10 controls, 9 *pdx1*^−/−^. Scale bar: 50 µm. (INL, inner nuclear layer; ONL, outer nuclear layer).

In the absence of enhanced cell production from the CMZ or activated Müller glia, rod progenitors remained as a potential source of new cells in *pdx1* mutants. To determine whether photoreceptors are replaced from the rod progenitor population in our diabetic mutants, we examined *pdx1* mutants expressing the neurod:GFP transgene. In the ONL of neurod:GFP transgenic controls at 6–10 mpf, we found either no or few rod-shaped strongly GFP+ cells (Figure 7a,b, black arrow), consistent with sporadic differentiation of new photoreceptors from rod progenitor cells. GFP fluorescence was otherwise weakly detected throughout the photoreceptor layer. In the ONL of *pdx1* mutants, we found rod-shaped GFP+ cells (Figure 7c, left panel, black arrow) and, additionally, cells with a different morphology resembling that of cones (Figure 7c, white arrow). We could distinguish between a phenotype with a multi-layer ONL and robustly GFP-labeled rod-shaped cells (Figure 7c, left panel) and a phenotype of severely reduced ONL thickness associated with both cone and rod-shaped GFP positive cells (Figure 7c, right panel, Table 2).

As the *neurod*-expressing rod progenitors originate in the INL, we compared the number of INL neurod:GFP+ cells in the mutants versus controls. In contrast to our finding no additional *pax6* expression in mutants, *pdx1* mutants did show a significant increase in neuro:GFP positive cells in the INL (Figure 7a,d). Although we did not detect enhanced INL proliferation in mutants, increased but still infrequent cell divisions occurring over the course of months may go undetected by the EdU assay. The duration of several days required for photoreceptor formation [41], combined with the stability of GFP, could lead to an accumulation of neurod:GFP expressing cells.

**Figure 8:**
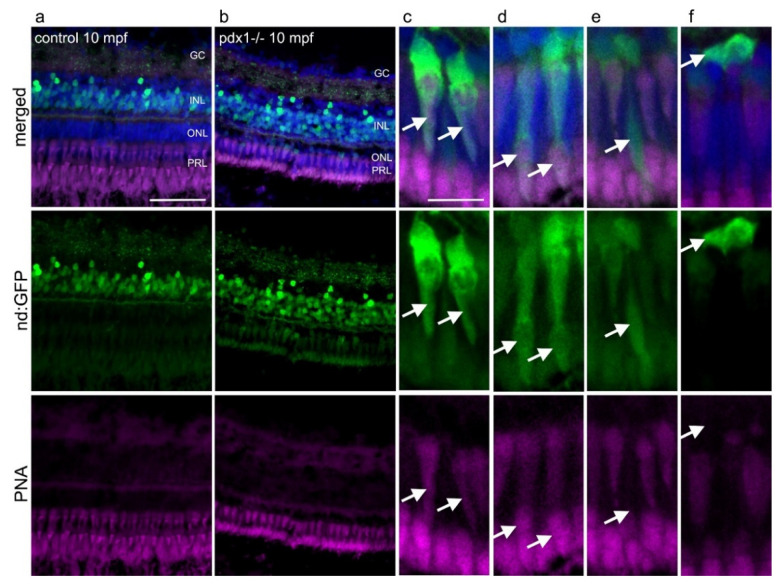
neurod:GFP expressing progenitors give rise to cones and rods in *pdx*^−^/^−^ diabetic zebrafish. Expression of GFP in tg(neurod:GFP) in the INL in control fish (**a**) and in the INL and in the ONL in *pdx1*^−/−^ fish (**b**). (**c**–**f**) Higher magnification views of photoreceptors from *pdx1*^−/−^ samples as in (**b**). GFP expression overlaps with PNA in cells with cone morphology (**c**,**d**, arrows), while rod-shaped cells (**e**, arrow) are PNA negative. Compact cells located basally in the ONL, presumed to be undifferentiated, do not label with PNA (**f**, arrow). Sections were counterstained with DAPI (blue). Scale bar: 50µm in a and b and 10µm in (**c**–**f**). (INL, inner nuclear layer; ONL, outer nuclear layer; PRL, photoreceptor layer).

To confirm the photoreceptor types arising from neurod:GFP-expressing cells, cones were labeled using fluorescently-tagged peanut agglutinin (PNA) [53]. PNA labeling indicated the aligned cones in controls (Figure 8a) and revealed the severely truncated cone outer segments of *pdx1* mutants (Figure 8b). In the *pdx1* mutants, GFP-positive cells were labeled by PNA, confirming their cone identify (Figure 8c,d). As PNA labels both inner and outer segments of cones [54], morphology of the labeled cells is further suggestive of cone subtype. We could distinguish cells with short outer segments consistent with a UV (SSC) cone morphology and cells with extended and clefted outer segments suggestive of RG double cones, although we did not verify RG double versus long single (blue) cone identity (Figure 8c,d, arrows). Intervening GFP+ cells not labeled by PNA have the elongated morphology of rods (Figure 8e, arrow). Compact GFP+ cells lacking PNA staining and located adjacent to the outer plexiform layer likely represent an earlier stage of differentiating photoreceptors [55], (Figure 8f).

Overall, these data are consistent with a restorative response to photoreceptor degeneration whereby progenitors normally restricted to a rod fate are partly redirected to also replenish the diminished cone population.

## 4. Discussion

Here we have analyzed injury responses to chronic hyperglycemia in the neural retina of the zebrafish diabetic *pdx1* mutant. While consequences of acute and chronic retinal injury have been described in zebrafish, our model of a chronic metabolic insult shows features not previously observed. As increased apoptosis was not detected, but photoreceptors showed disrupted morphology and reduced cell numbers, we considered hyperglycemia to cause a low-grade injury. In contrast to acute injury models, we generally did not observe enhanced proliferation in the INL. An increase in neurod:GFP positive cells in the INL and the appearance of neurod:GFP positive rod and cone cells in the ONL points to a response providing for partial restoration of lost rods and cones in *pdx1* mutants. This photoreceptor replacement does not seem to involve signaling and transcription factors previously reported in models assessing regeneration after acute injury, such as Notch signaling, GFAP upregulation or expression of *pax6*. Surprisingly, photoreceptor progenitor cells considered to be rod-lineage restricted were apparently directed to generate replacement cones as well as rods.

Notch signaling is an essential pathway for regulating cell fate decisions in development and regeneration in zebrafish retina [11]. Expression analysis showed upregulation of Notch–Delta pathway components in the INL upon thermal or mechanical injury [12,47]. In other studies, Notch signaling was reported to be active in resting Müller glial cells using the Tp1:hmgb1-mCherry transgene, and this signal decreased upon retinal injury and subsequent Müller glia proliferation [11,56]. Studies in Medaka, in which expression was compared between a normal and a destabilized Notch signaling reporter, revealed a similar result to ours [46], namely, active signaling during Müller glia specification that is downregulated as development proceeds. The disparity can be explained by considering that the nuclear-localized hmgb1-mCherry transgene is extremely stable, and so may reflect previous and not ongoing Notch signaling activity, and becomes diluted by cell proliferation. This is supported by our finding of co-expression of the GFP and mCherry reporters early in development, followed by progressive loss of GFP, with maintenance of mCherry in a limited cell population within the INL. As we showed overlapping Tp1:GFP/Tp1:mCherry expression in vascular tissues in adults, the Tp1:GFP transgene is responsive at later stages. However, we cannot exclude tissue-specific silencing of the Tp1:GFP transgene within the retina. Further examination of Notch responses using alternative reporter lines and interrogation of Notch ligands, receptors and downstream targets will help to clarify this issue.

The adult zebrafish retina contains two stem cell populations that contribute new neurons throughout the zebrafish lifespan: the ciliary marginal zone (CMZ) and Müller glial cells located in the INL [12]. The multipotent stem cells of the CMZ generate all neurons except rod photoreceptors, while rod photoreceptors emerge from progeny of sporadically dividing Müller glia [49]. Proliferation in the CMZ of *pdx1* mutants was similar to controls during young adult stages, and showed similar decline with aging between 3 mpf and 6 mpf. At 10 mpf, *pdx1* mutants showed reduced proliferation in comparison to controls. Proliferative decline of neural stem cell populations with aging has been previously documented across vertebrate species [57]. Due to the poor survival of the mutants beyond 12 mpf, we cannot address the consequences of these proliferative changes for retinal maintenance in later adult stages. Our results are further consistent with reports that chronic hyperglycemia disrupts neurogenesis and negatively impacts the maintenance of neural stem and progenitor cells [58,59].

The mechanisms by which hyperglycemia perturbs stem cell proliferation are poorly defined, but may be an adverse effect from disrupted barrier function changing the extracellular milieu with increased exposure to inflammatory cytokines, or it could result from alterations in insulin signaling pathways [58,60]. Although we did not see immune cell infiltration into the retina, this does not rule out an enhanced inflammatory state. Accumulating perivascular microglia release cytokines that can penetrate into the retina [21], and retinal neurons themselves, including photoreceptors, can secrete pro-inflammatory factors [7]. The elevated expression of the inflammatory mediator NF-KB was detected by Western blot in retinal homogenates from zebrafish following four weeks of alternating glucose immersion, but the cell of origin was not identified [24]. Levels of inflammatory mediators within the retinas of *pdx1* mutants remain to be investigated.

In our diabetic model, we detected the most striking pathology within the photoreceptor layers, consisting of truncation of outer segments and cell loss. Photoreceptors are particularly susceptible to disturbances of glucose regulation due to their high metabolic activity and their primary use of glucose as an energy source [61]. In diabetes, altered expression or activity of glucose transporters can lead to an imbalance of energy supply and demand within the retina [62]. Byproducts of excess glucose metabolism and elevation of reactive oxygen species cause chronic injury to neurons and the vasculature [63]. It was unexpected that we failed to detect TUNEL labeled cells to correlate with photoreceptor loss. However, cell death may be under-detected in our studies, as TUNEL only indicates events occurring at the discrete time point of tissue harvest, and the degeneration proceeds over many months. Furthermore, cell death of retinal neurons can occur by several different mechanisms besides apoptosis, including necrosis and pyroptosis, and these may be better detected by alternative methods [64,65]. Photoreceptor loss becomes apparent between 3 mpf and 6 mpf. Within this time window, the precise initiating factors remain to be defined. This model will allow identification of these triggers and investigation into factors that promote neuroprotection and photoreceptor restoration, and thereby help to define novel therapeutic approaches for early stage disease.

In mammalian retina, Müller glia are an important component of the injury response, which is initially neuroprotective. However, persistent activation, as occurs in diabetic retinopathy, is accompanied by diminished Müller glia function and uncontrolled proliferation. Excess numbers of disorganized cells, combined with production of fibrosis-promoting factors, blocks restoration of normal tissue architecture and leads to retina scarring, detachment and blindness [66]. By contrast, in zebrafish acute injury models, a proliferative response by Müller glia is associated with neuronal regeneration and tissue restoration. The Müller glia response in our disease model is distinct in showing upregulation of glutamate synthetase [26], no change in GFAP expression and an absence of proliferation. While not actively proliferating, the slow cell cycling characteristic of Müller glia appeared to be maintained, as the progeny of these cell divisions are the rod progenitor population, which continued to generate replacement photoreceptors after 10 months of disease progression.

Until recently, studies of retina regeneration in zebrafish have primarily focused on recovery from acute injuries, in which a robust proliferative response from Müller glia is followed by replacement of the missing cell types [9]. By contrast, this acute response is absent when the insult is chronic or does not reach a threshold of severity. Instead, precursor proliferation in the ONL that restores lost rod photoreceptors has been reported in a low dose nitroreductase-induced rod ablation model [67] and in a low-intensity light injury model [68]. We suggest that the slowly progressing cell loss in *pdx1* mutants is insufficient to activate Müller glial proliferation.

**Figure 9:**
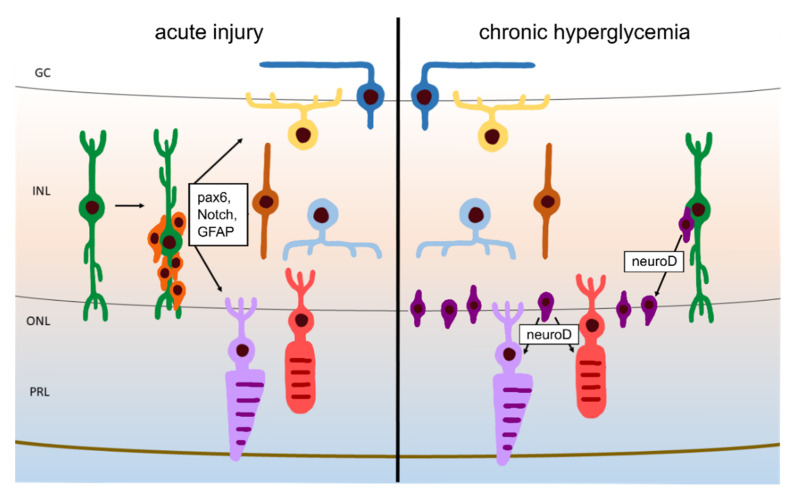
Our proposed model of regeneration during chronic hyperglycemia versus regeneration upon acute injury. Following acute injury, activated Müller glia divide to produce multipotent progenitors that can give rise to all neuronal cell types. Under conditions of chronic hyperglycemia, lost photoreceptors are replenished through *neurod*-expressing progenitors that likely arise in the INL. Unlike the classically described “rod progenitors”, these proliferative progenitors give rise to both rods and cones.

The injury response to chronic hyperglycemia characterized here is distinct in the apparent replacement of cones from the ‘rod progenitor’ cell population, as we observed neurod:GFP+ cones in the ONL of *pdx1* mutants which were not seen in controls (Figure 9). Since we did not detect additional *pax6* expression, we hypothesize that increased *neurod*-expressing cells in the INL arise from committed photoreceptor precursors, and not from a highly proliferative multipotent progenitor population. We propose that our EdU-labeling method was not sensitive enough to reveal an increase in mitosis in a slowly amplifying population. Detection of progenitor proliferation in the INL requires long and repeated exposures to nucleotide analogs [9], which we did not achieve with our single injection protocol. As rod and cone progenitors share highly similar transcriptional programs, the observed fate plasticity is biologically realistic [49,50,69] and has been proposed to contribute to cone regeneration in a larval UV cone-ablation paradigm [70].

The ONL proliferation observed at 6 mpf fell into two categories, low versus high, and a similar partition was observed for the occurrence of samples with neurod:GFP+ cones. It has been suggested that extracellular signals and cell–cell interactions regulate photoreceptor fate allocation in the ONL [49,70]. It is possible that alterations in the signaling milieu triggered by severe cell loss in the ONL drive enhanced proliferation, in coordination with diversion of some cells to a cone fate. Identification of the responsible signaling processes is made challenging by the lack of a distinct marker for rod progenitors, but perhaps the cell lineages are more fluid than previously thought.

While similarities to human DR can be identified in the diabetic zebrafish model used here, there are important differences to note when considering the relevance for deciphering mechanisms of DR disease pathology. Foremost is the regenerative capacity of retinal neurons in the zebrafish [11]. As we have now shown that a regenerative response occurs under persistent hyperglycemic conditions, injury responses are therefore different from mammals in some respects, and efforts to define disease progression become complicated. However, our model provides a system to identify regenerative mechanisms that are robust to metabolic dysregulation. Another disadvantage relates to the small size of the zebrafish, which is advantageous for maintenance, but poses challenges for performing biochemical and metabolic analyses [71]. Whereas in rodents, glucose levels can be measured repeatedly in the same animals, our diabetic animals do not tolerate this. Therefore, we cannot monitor variations in glycemia occurring over time to help us understand the source of the phenotypic variability we see.

## 5. Conclusions

In conclusion, this study revealed that diabetic retinopathy in the *pdx1* diabetic zebrafish features not only neural pathologies, but also a restorative response based on the enhanced regenerative properties of the zebrafish retina. Although long considered unable to regenerate, recent studies have demonstrated that mammalian Müller glial cells possess a dormant regenerative potential [72]. Deciphering the distinct regenerative process activated in zebrafish retina under diabetic conditions may help to define pathways that can be targeted in humans for neuroprotection and to promote repair of tissue injuries.

## Figures and Tables

**Table 1:** Double positive pax6:dsRed, EdU stained cells in the CMZ per section (at least 3 sections per individual, *n* = 2 fish per time point).

	3 mpf	6 mpf
Control	3.5	0.6
*Pdx1* ^−/−^	3.6	0.4

**Table 2:** Frequency of samples with a field of view showing rod and/or cone shaped GFP+ cells in the ONL.

	Rod Shaped GFP+ Cells	Rod and Cone Shaped GFP+ Cells
6–10 mpf control	70% (7/10)	0% (0/10)
6–10 mpf *pdx1*^−/−^	67% (6/9)	89% (8/9)

## Data Availability

Material and Data pertaining to this manuscript are available from the corresponding author pending reasonable request.

## Supplementary Materials

The following are available online at https://www.mdpi.com/article/10.3390/cells10113265/s1, Figure S1: Sustained high glucose levels in *pdx1*^−/−^ mutant zebrafish, Figure S2: Reduced cells in the ONL of *pdx1* mutants, Figure S3: Variable proliferation phenotypes in *pdx1*^−/−^ mutant neural retina, Figure S4: GFAP staining is not changed by chronic hyperglycemia, Figure S5: Cell populations labeled by pax6b:dsRed and neurod:GFP.
