# Supplementary material for: Differential Responses of Neural Retina Progenitor Populations to Chronic Hyperglycemia
Source: Cells. 2021 Nov 22;10(11):3265. doi: 10.3390/cells10113265 (PMC8622914; doi:10.3390/cells10113265)
Supplement: Supplementary file 1 [file cells-10-03265-s001.zip › cells-1423390-supplementary.pdf]

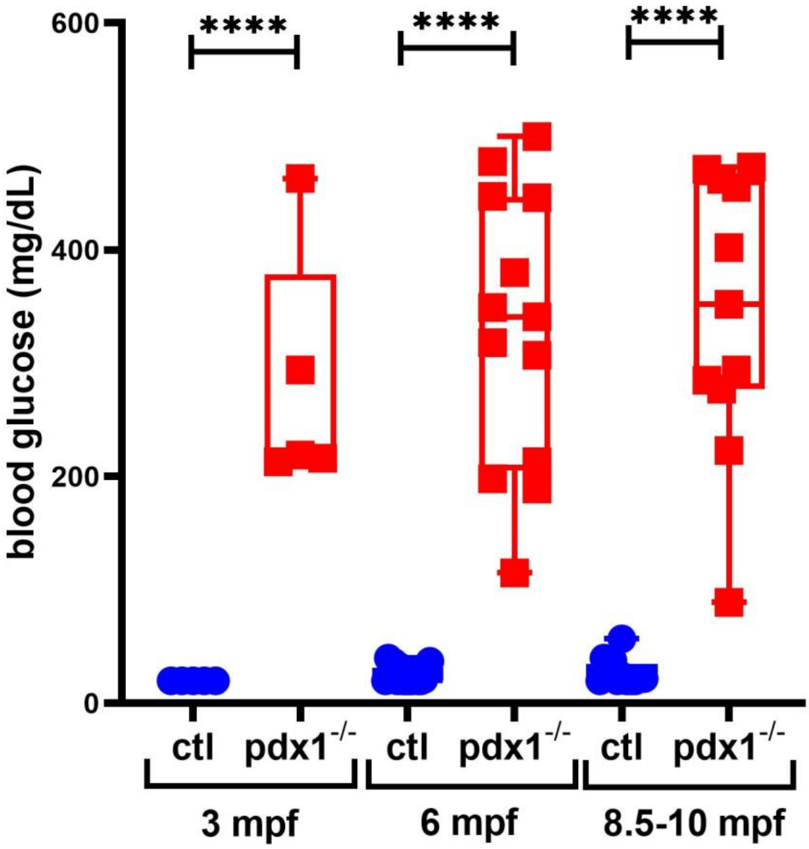

Figure S1. Sustained high glucose levels in *pdx1*$^{-/-}$ mutant zebrafish. Blood glucose levels measured just prior to tissue harvest. Box plot extends from 75% to 25%, showing all data points, line indicates median; n= 38 control animals and 30 *pdx1* mutants (9 control, 5 *pdx1* mutants at 3 mpf, 17 controls and 14 *pdx1* mutants at 6 mpf and 12 controls and 11 mutants at the age of 8 to 10 mpf). ****P<0.0001(t-test).

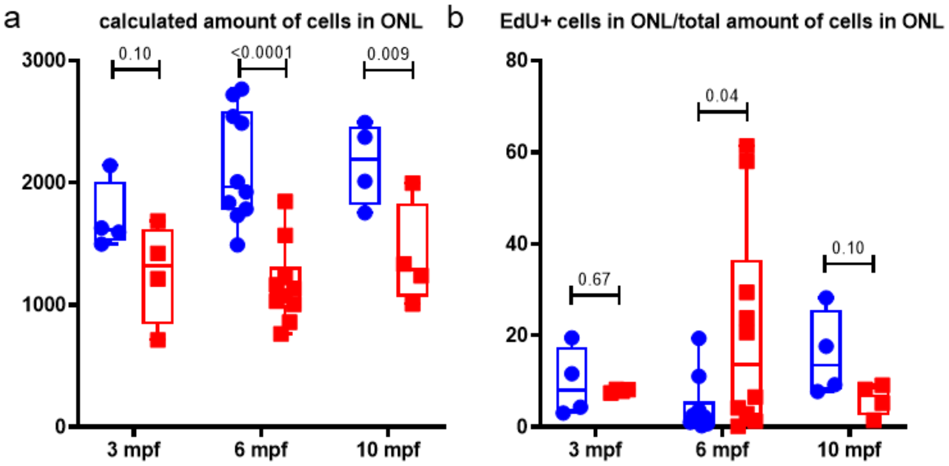

Figure S2. Reduced cells in the ONL of *pdx1* mutants. (a) Graph displaying the estimated cell number in the ONL. Cells were counted in 50µm linear regions of the ONL and the amount was extrapolated to the whole length of the retina. (b) The values from (a) were used to calculate the proportion EdU positive cells in the ONL. Box plot extends from 75% to 25%, showing all data points, line indicates median; n= 4-7 fish per time point.

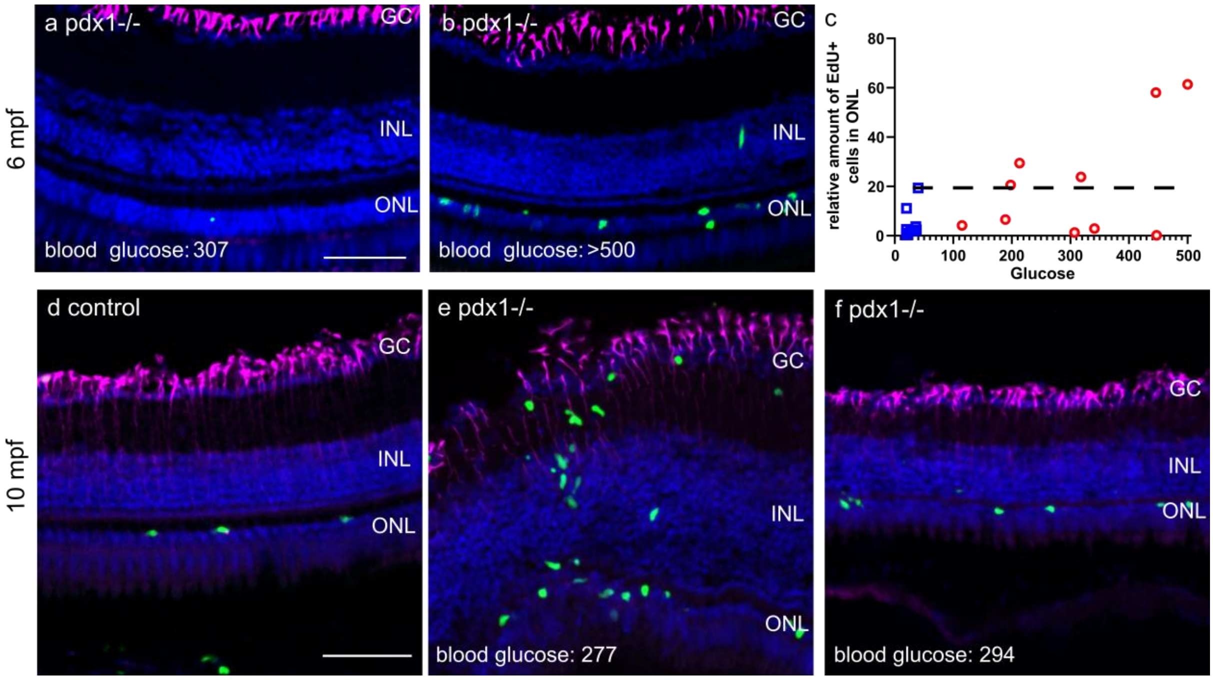

Figure S3. Variable proliferation phenotypes in *pdx1-/-* mutant neural retina. (a, b) Cryosections of 6 mpf *pdx1* mutant retinae showing variable phenotypes with respect to the number of EdU positive (green) proliferative cells in the ONL (a, low proliferation, n=5; b, enhanced proliferation, n=5). GFAP staining (magenta) was not enhanced in either condition. (c) There was no significant correlation between blood glucose levels and the number of EdU positive cells in the ONL (r = 0.53, p = 0.11, n= 10). (d-f) 10 mpf control (d) and *pdx1-/-* mutant (e, f) fish displayed mainly proliferation in the ONL, as shown for younger stages, with the exception of one *pdx1-/-* mutant fish (n=4) which showed many EdU positive cells in the IPL and INL. GFAP staining was not increased. Scale bar: 50µm. (GC, ganglion cell layer; INL, inner nuclear layer; ONL, outer nuclear layer).

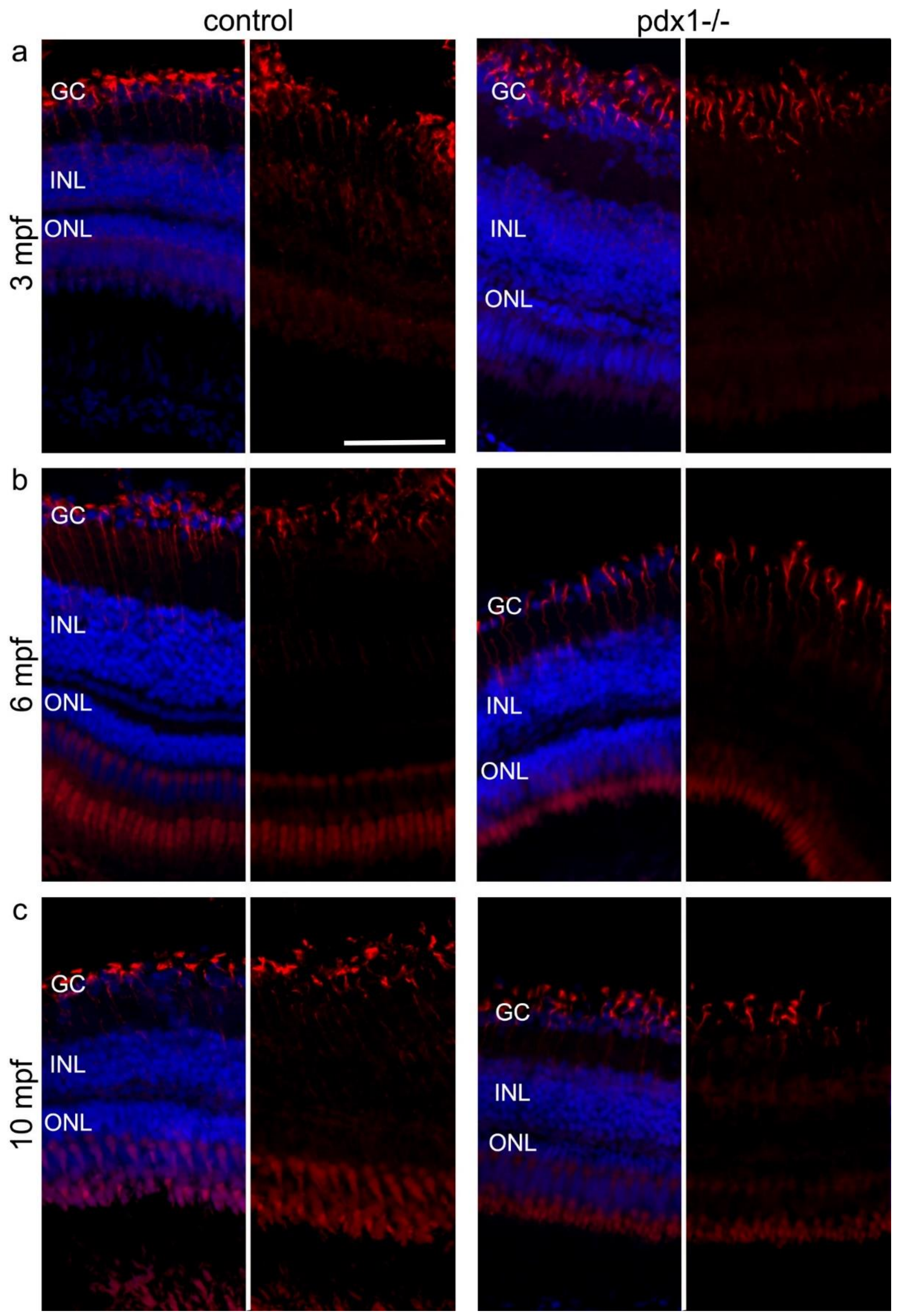

Figure S4: GFAP staining is not changed by chronic hyperglycemia. Retinal cryosections were stained with GFAP (red) and counterstained by DAPI. GFAP staining of Müller glia cells was similar between controls and *pdx1* mutants at 3 mpf (a), 6 mpf (b) and 10 mpf (c). Scale bar: 50µm. (GC, ganglion cell layer; INL, inner nuclear layer; ONL, outer nuclear layer).

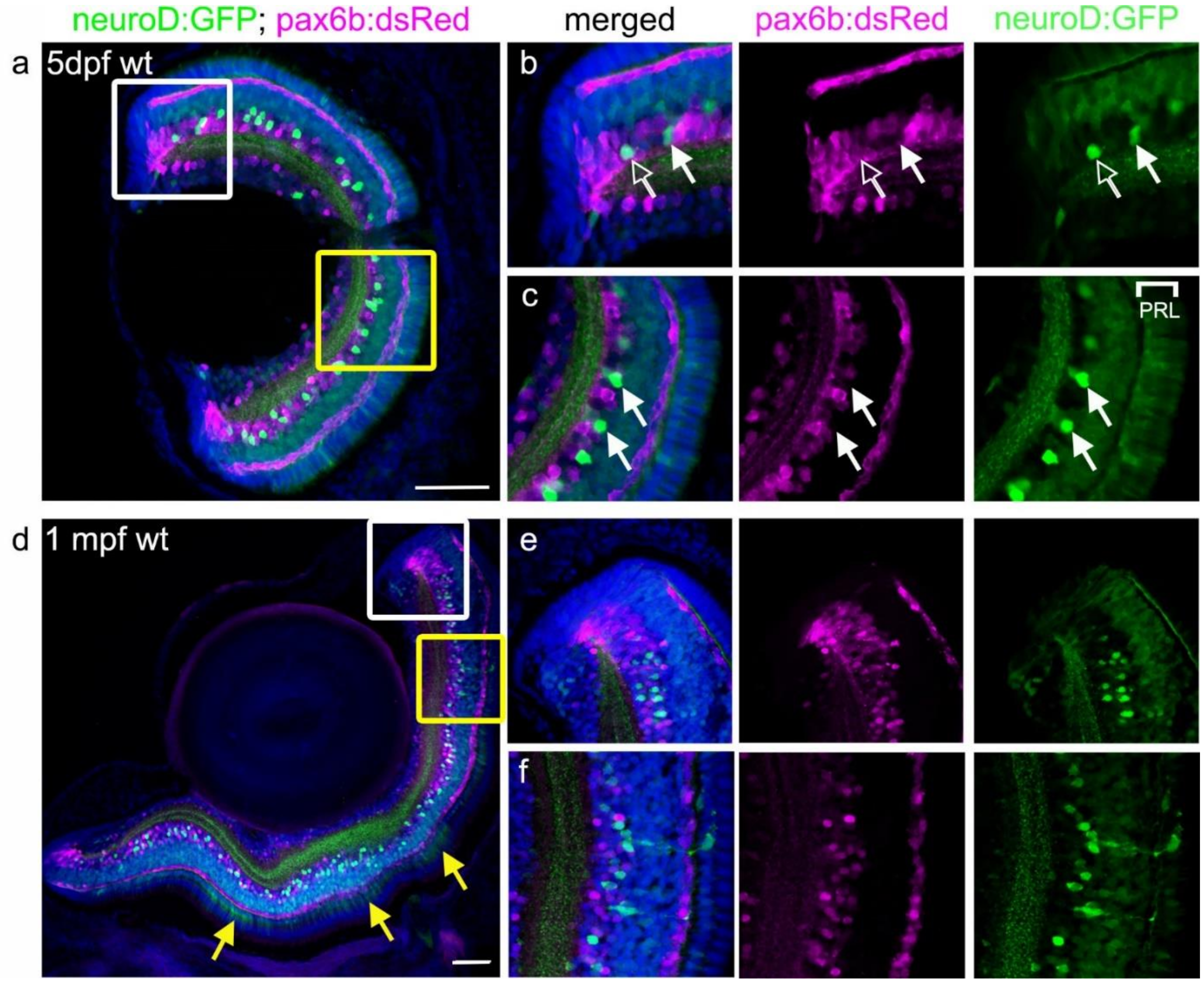

Figure S5. Cell populations labeled by pax6b:dsRed and neurod:GFP. (a-c) In wild types at 5 dpf, pax6b:dsRed labels clusters of cells next to the stem cells niche in the CMZ (b). In the central part (c) of the retina, dsRed is expressed in cells of the ganglion cell layer, the upper INL, and the lower INL (presumably horizontal cells). neurod:GFP positive cells with strong GFP expression are found in the basal INL, while weak GFP expressing cells are in the apical INL and in the photoreceptor layer (PRL). (d-f) In 1 mpf old fish the expression pattern of pax6b:dsRed is similiar to 5 dpf. Neurod:GFP expression still shows strong and weak expressing cells in the INL. GFP expression is also found in the photoreceptor layer (d, yellow arrows) and occasionally cells with strong GFP expression and long processes crossing from the INL to the ONL are observed (f). Scale bar: 50µm.
